# Sleep disorders, mental health, and dry eye disease in South Korea

**DOI:** 10.1038/s41598-022-14167-0

**Published:** 2022-06-30

**Authors:** Youngju An, Hyojin Kim

**Affiliations:** 1 Department of Optometry, Baekseok Culture University, Cheonan, 31065 Korea; 2 Department of Optometry, Division of Health Science, and Graduate School of Health and Welfare, Baekseok University, Cheonan, 31065 Korea

**Keywords:** Eye diseases, Quality of life

## Abstract

Dry eye disease (DED) is a multifactorial disease of the ocular surface causing severe discomfort, mild ocular irritation, fatigue, pain, visual disturbance, and foreign body sensation. Stress, depression, and sleep disorders are risk factors for DED. We aimed to investigate the association between DED symptoms and composite factors related to mental health (combined sleep duration, psychological stress perception, and history of depressed mood) in Korean adults aged ≥ 20 years in a population-based study using the 2010–2012 Korea National Health and Nutrition Examination Survey data. Symptoms of DED and data on mental health were obtained using questionnaires. Multiple logistic regression analysis was conducted to examine the association between mental health and DED, and adjusted for possible covariates. Subjects with symptoms of DED were more likely to experience short sleep duration, psychological stress perception, and a history of depressed mood [odds ratio (OR) = 1.42, 95% confidence interval (CI) 1.06–1.90; OR = 1.71, 95% CI 1.37–2.14; and OR = 1.37, 95% CI 1.06–1.77, respectively] even after correcting for demographic factors, lifestyle factors, and medical factors. Additionally, participants with symptoms of DED were more likely to experience composite factors related to mental health (OR = 1.91, 95% CI 1.07–3.39). Therefore, ophthalmologists may report difficulties in both sleep and mental health in patients with DED.

## Introduction

Dry eye disease (DED) is a growing public health problem in ophthalmology^1^. It is defined as a multifactorial disease of the tear ducts and ocular surface, accompanied by increased osmolality of the tear film and inflammation of the ocular surface^2^. The prevalence of dry eye disease among middle-aged and elderly individuals ranges from 7 to 34%^3^. The incidence of DED, which is one of the most common ophthalmic diseases, has been markedly increasing in industrialized countries^4^.

DED results in severe discomfort, mild ocular irritation, fatigue, pain, visual disturbance, foreign body sensation, and tear film instability, all of which can potentially damage the ocular surface^2,5,6^. It interferes with activities of daily living^7^, thus negatively affecting patients’ mental health and productivity at work^8,9^. DED is recognized as a major public health problem worldwide^6^, and it can place a significant financial burden on the healthcare system^10,11^.

Several studies have reported the risk factors for DED. These risk factors included old age, female sex, smoking status, wearing contact lenses, systemic medications, video display use, and a history of ocular surgery^3,7,12–15^. Depression, sleep disorders, and stress are also considered risk factors for DED, based on recent reports^9,16,17^. Recently, there has been increased medical interest in the psychiatric pathophysiology of patients with DED^18^.

We hypothesized that there would be a complex effect in DED affected by sleep and mental health on DED. To the best of our knowledge, relatively few studies have investigated whether DED affects sleep duration or psychological stress perception. Therefore, the present study aimed to determine the relationship between DED and composite factors related to mental health (combined sleep duration, psychological stress perception, and history of depressed mood) in a representative sample of Korean adults aged ≥ 20 years, using data from the 2010 to 2012 Korea National Health and Nutrition Examination Survey (KNHANES).

## Results

**Figure 1:**
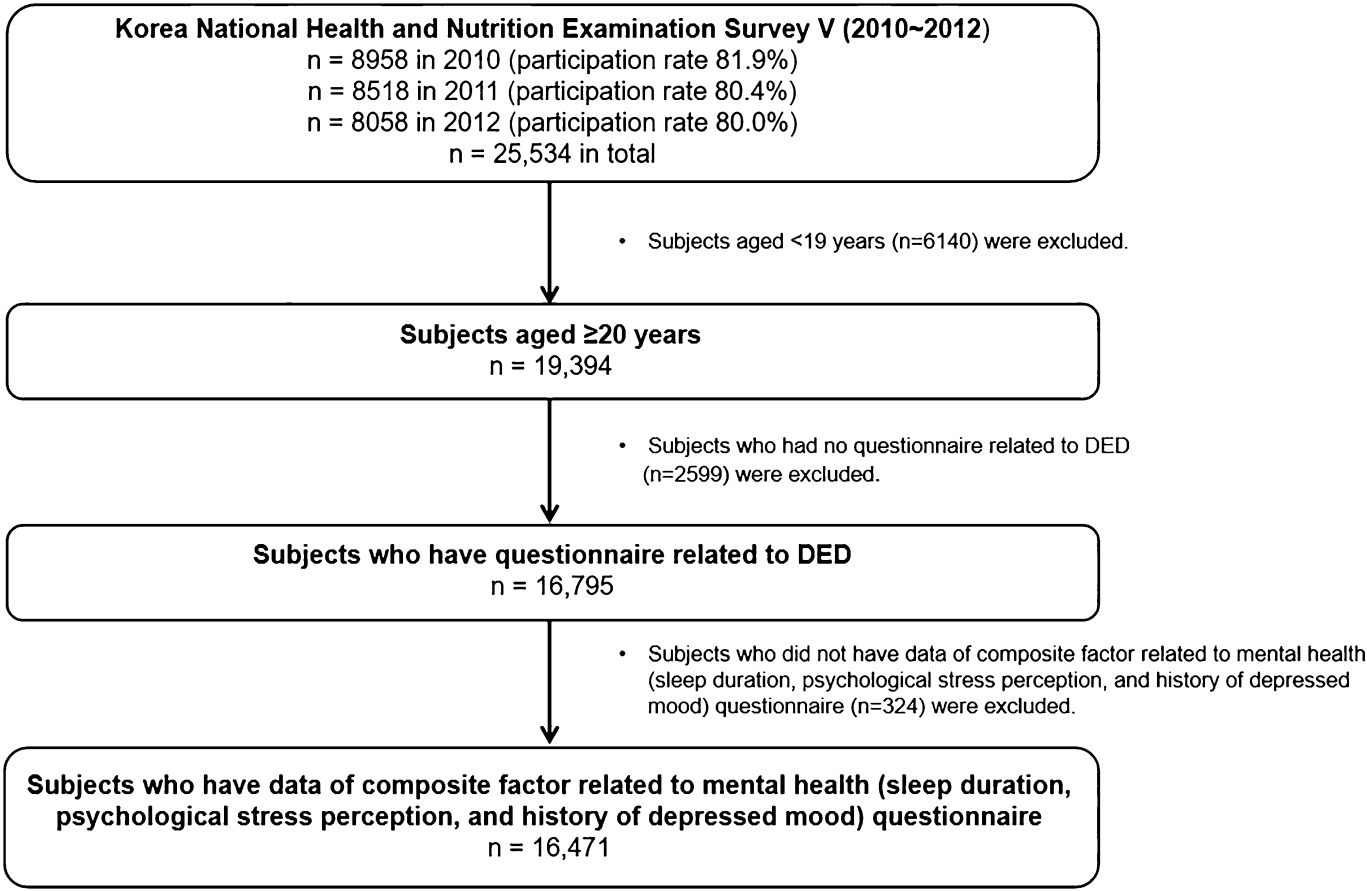
Flow chart of the study population.

Among the 25,534 people who participated in KNHANES V (2010–2012), 19,394 participants were aged ≥ 20 years. The, 2599 and 324 participants lacked data on symptoms of DED and mental health-related characteristics, respectively, and were excluded from the study, leaving a final number of 16,471 participants (Fig. 1).

In adults aged ≥ 20 years, the weighted prevalence of symptoms of DED (95% confidence interval [CI]) was 16.19% (15.09–17.29), with a prevalence of 10.61% (9.49–11.73) among men and 21.55% (20.07–23.03) among women (data not shown).

**Figure 2:**
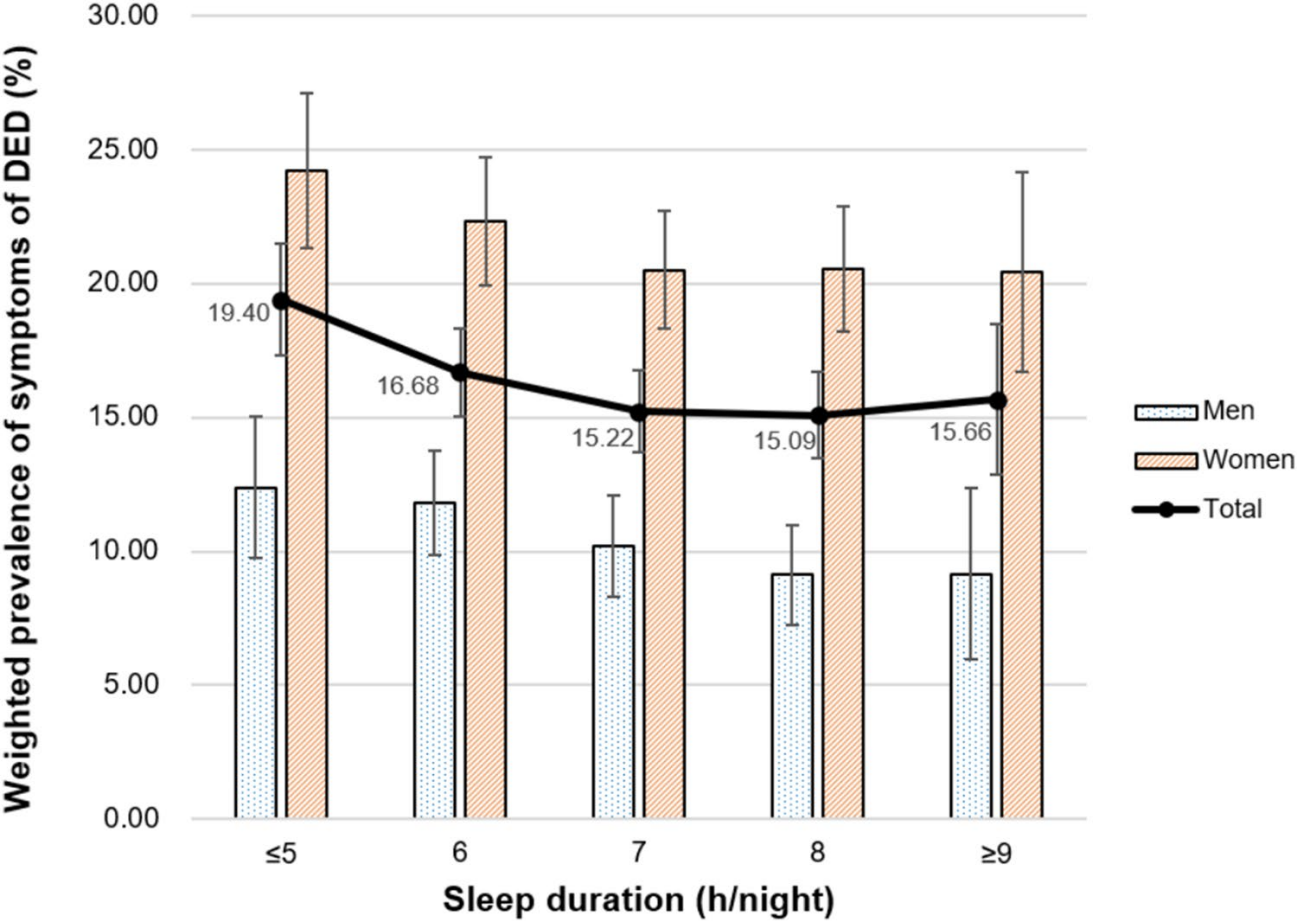
Relationship between sleep duration and weighted prevalence of symptoms of DED in Korean adults. Error bars represent 95% confidence intervals.

**Figure 3:**
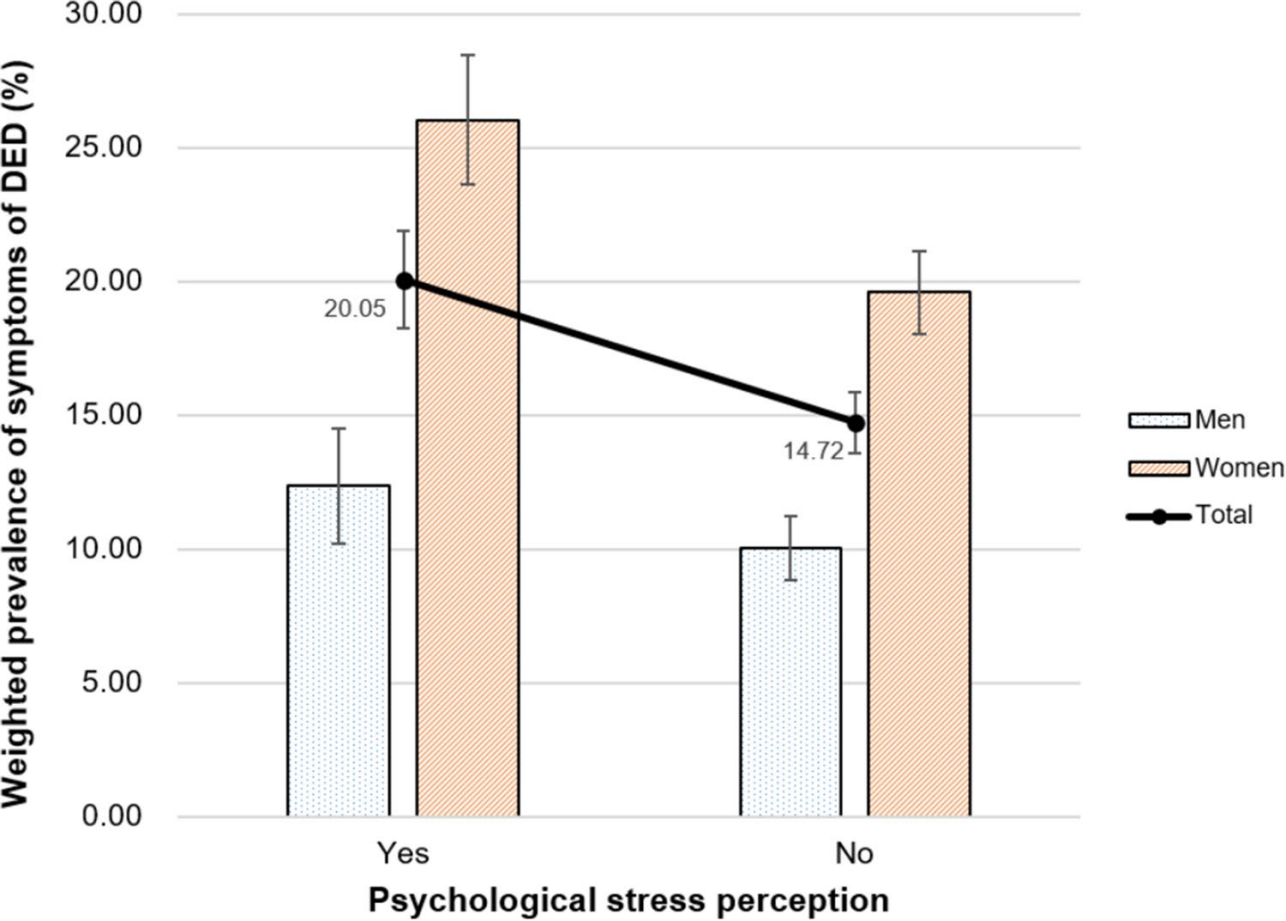
Relationship between psychological stress perception and weighted prevalence of symptoms of DED in Korean adults. Error bars represent 95% confidence intervals.

**Figure 4:**
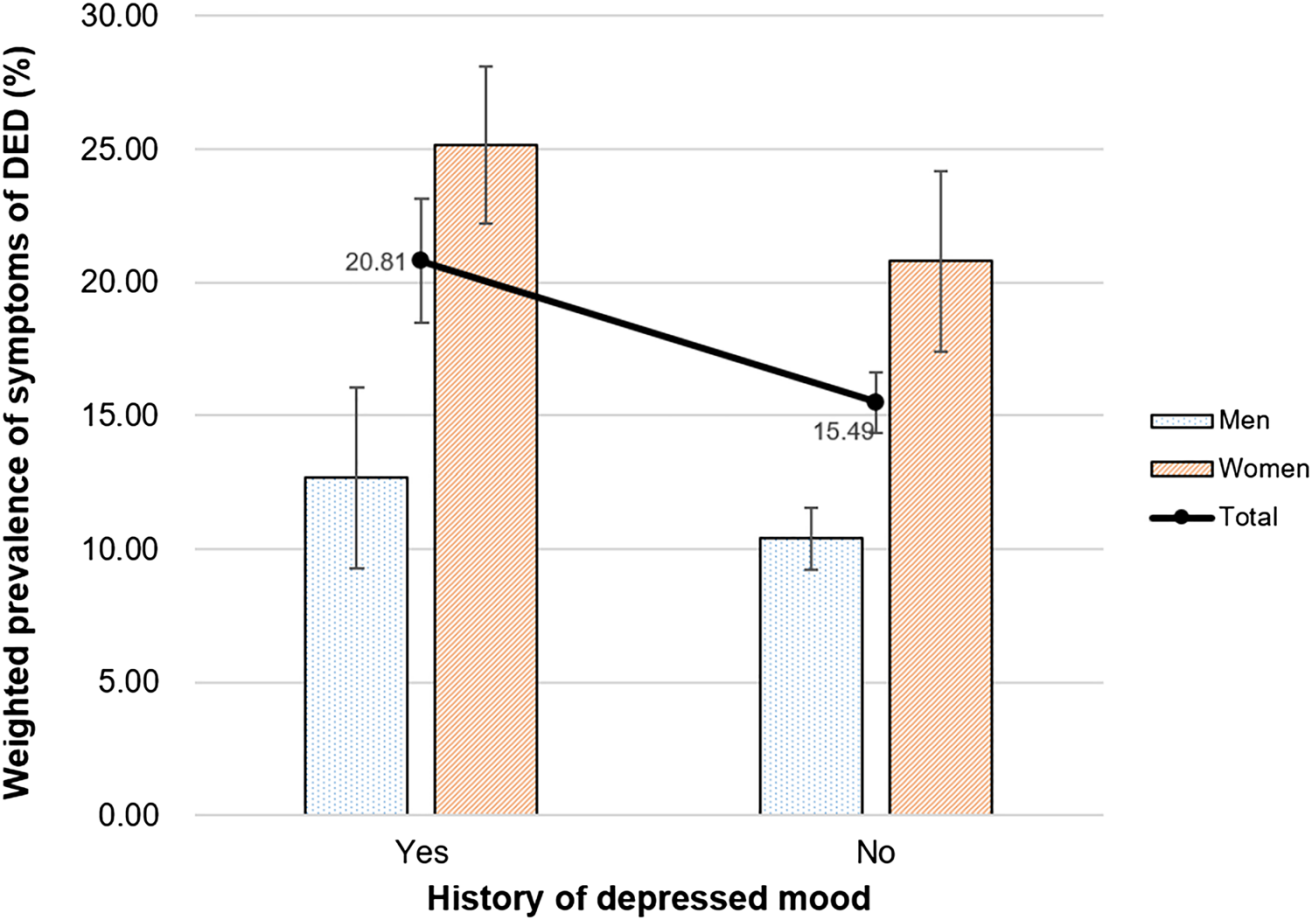
Relationship between history of depressed mood and weighted prevalence of symptoms of DED in Korean adults. Error bars represent 95% confidence intervals.

**Figure 5:**
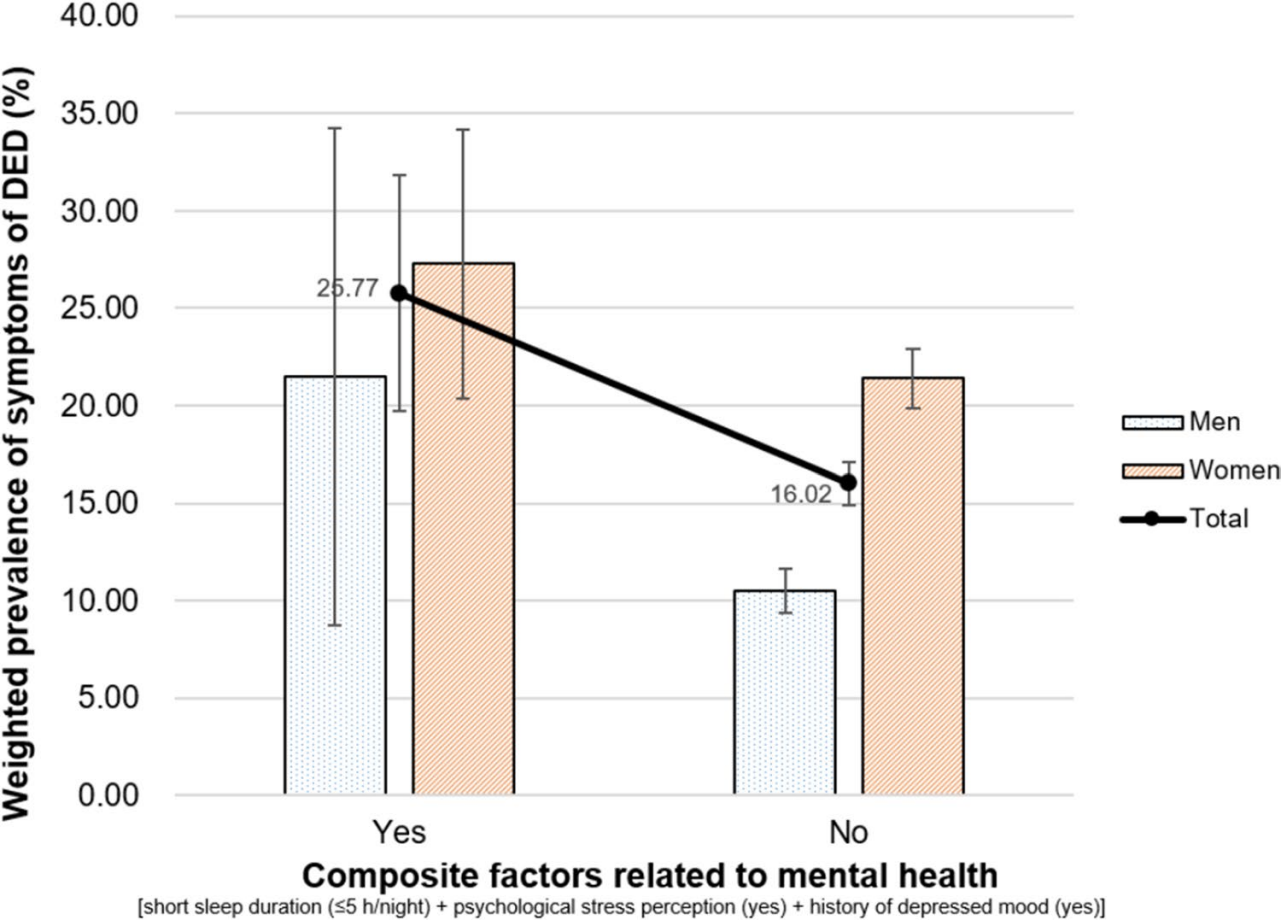
Relationship between composite factors related to mental health [sleep duration (≤ 5 h/night) + psychological stress perception (yes) + history of depressed mood (yes)] and weighted prevalence of symptoms of DED in Korean adults. Error bars represent 95% confidence intervals.

The relationship between mental health-related characteristics and the weighted prevalence of DED symptoms is presented in Figs. 2, 3, 4 and 5. The weighted prevalence of symptoms of DED (95% CI) was 19.40% (17.36–21.43), 16.68% (15.07–18.28), 15.22% (13.72–16.73), 15.09% (13.49–16.68), and 15.66% (12.89–18.43) for participants with sleep durations ≤ 5, 6, 7, 8, and ≥ 9 h/night, respectively (Fig. 2). The weighted prevalence of DED symptoms (95% CI) was 20.05% (18.25–21.85) in those with psychological stress perception (Fig. 3) and 20.81% (18.52–23.11) in those with a history of depressed mood (Fig. 4).

The relationship between composite factors related to mental health [combined short sleep duration (≤ 5 h/night), psychological stress perception (yes), and history of depressed mood (yes)] and weighted prevalence of DED symptoms are presented in Fig. 5. The weighted prevalence of DED symptoms (95% CI) was 25.77% (19.80–31.73) and 16.02% (14.92–17.11) among those with and without composite factors related to mental health, respectively.

Demographics, lifestyle, and medical characteristics of the participants are shown in Table 1. Regarding demographic characteristics, we found that in comparison to those without DED symptoms, there were many cases with DED symptoms among participants aged < 30 years or ≥ 50 years, women, those with less than elementary school level education, college students, white-collar workers and those with no occupation. Regarding lifestyle characteristics, there were more cases with DED symptoms among those with underweight or normal BMI, current nonsmokers, those not exercising regularly, and participants with daily sun exposure of ≤ 2 h/day. Regarding the clinical characteristics, there were more cases with DED symptoms among pregnant participants, those with thyroid disease, and those with previous ocular surgery.

**Table 1:** Demographic, lifestyle, and medical characteristics between subjects with and without symptoms of DED (column %).

Characteristic	Overall (N = 16,471)		Symptoms of DED				*P* value^c^
			Yes (n = 2940)		No (n = 13,531)		
	n^a^	%^b^	n^a^	%^b^	n^a^	%^b^	
**Age (years) (n = 16,301)**							0.024
20–29	1727	18.1	315	18.3	1412	18.0	
30–39	3015	20.9	495	19.4	2520	21.2	
40–49	2890	21.9	451	19.9	2439	22.2	
50–59	3194	18.7	584	19.5	2610	18.6	
60–69	2857	10.9	573	12.8	2284	10.6	
≥ 70	2618	9.5	493	10.0	2125	9.4	
**Gender (n = 16,471)**							< 0.001
Men	6937	49.0	797	32.1	6140	52.2	
Women	9534	51.0	2143	67.9	7391	47.8	
**Education (n = 16,434)**							0.017
Elementary school	4293	19.1	841	21.5	3452	18.7	
Middle school	1801	10.2	297	9.6	1504	10.4	
High school	5459	38.9	944	36.3	4515	39.3	
University or higher	4881	31.8	848	32.6	4033	31.6	
**Household income (n = 16,282)**							0.523
Lowest quartile	3189	16.0	583	16.3	2606	15.9	
2nd quartile	4205	27.2	761	27.4	3444	27.2	
3rd quartile	4436	29.2	757	27.6	3679	29.5	
Highest quartile	4452	27.6	803	28.6	3649	27.4	
**Occupation (n = 16,399)**							< 0.001
No occupation	6762	35.8	1399	41.4	5363	34.7	
Blue collar	4280	26.7	607	20.9	3673	27.9	
White collar	5357	37.5	922	37.8	4435	37.4	
**Body mass index (n = 16,343)**							< 0.001
Underweight	747	4.9	160	6.1	587	4.6	
Normal	10,388	62.9	1923	66.6	8465	62.2	
Obesity	5208	32.3	835	27.3	4373	33.2	
**Current smoker (n = 16,469)**							< 0.001
Yes	3338	26.2	391	18.2	2947	27.8	
No	13,131	73.8	2549	81.8	10,582	72.2	
**Regular exercise (n = 16,471)**							0.016
Yes	6229	38.9	1065	36.2	5164	39.5	
No	10,242	61.1	1875	63.8	8367	60.5	
**Daily sun exposure (h/day) (n = 16,442)**							0.011
≤ 2	10,431	62.4	1950	65.9	8481	61.7	
2–5	3833	25.1	642	22.9	3191	25.5	
≥ 5	2178	12.5	344	11.2	1834	12.8	
**Pregnancy (n = 3175)**							0.047
Yes	28	1.0	8	1.8	20	0.8	
No	3147	99.0	865	98.2	2282	99.2	
**Diabetes (n = 16,442)**							0.705
Yes	1434	6.5	265	6.7	1169	6.5	
No	15,008	93.5	2669	93.3	12,339	93.5	
**Hypertension (n = 16,442)**							0.098
Yes	3899	17.7	757	19.1	3142	17.5	
No	12,543	82.3	2176	80.9	10,367	82.5	
**Thyroid disease (n = 16,440)**							< 0.001
Yes	680	3.3	189	5.8	491	2.8	
No	15,760	96.7	2745	94.2	13,015	97.2	
**Rheumatoid arthritis (n = 16,440)**							0.072
Yes	418	1.9	90	2.4	328	1.8	
No	16,022	98.1	2843	97.6	13,179	98.2	
**History of ocular surgery (n = 16,308)**							< 0.001
Yes	2417	11.7	662	19.5	1755	10.2	
No	13,891	88.3	2232	80.5	11,659	89.8	

^a^Unweighted sample size.

^b^Weighted percentage.

^c^Weighted *P* value.

The mental health-related characteristics of the subjects with and without symptoms of DED are shown in Table 2. Compared with participants without symptoms of DED, those with symptoms of DED frequently reported a sleep duration of ≤ 5 h/night or ≤ 6 h/night, as well as psychological stress perception, history of depressed mood, and composite factors related to mental health.

**Table 2:** Mental health related characteristics between subjects with and without symptoms of DED (column %).

Characteristic	Symptoms of DED				*P* value^c^
	Yes (n = 2940)		No (n = 13,531)		
	n^a^	%^b^	n^a^	%^b^	
**Sleep duration (h/night)**					0.002
≤ 5	563	16.4	2028	13.2	
6	756	27.7	3527	26.8	
7	781	26.7	3848	28.7	
8	626	21.5	3045	23.4	
≥ 9	214	7.7	1083	8.0	
**Psychological stress perception**					< 0.001
Yes	933	34.2	3420	26.3	
No	2007	65.8	10,111	73.7	
**History of depressed mood**					< 0.001
Yes	512	16.9	1725	12.5	
No	2428	83.1	11,806	87.5	
Composite factors related to mental health*					< 0.001
Yes	88	2.8	248	1.6	
No	2852	97.2	13,283	98.4	

^a^Unweighted sample size.

^b^Weighted percentage.

^c^Weighted *P* value.

*Composite factors related to mental health; short sleep duration (≤ 5 h/night) + psychological stress perception (yes) + history of depressed mood (yes).

Table 3 shows the results of multiple logistic regression analyses of the association between DED symptoms and related mental health characteristics. Subjects with symptoms of DED were more likely to experience short sleep duration, psychological stress perception, and a history of depressed mood [odds ratio (OR) = 1.42, 95% CI 1.06–1.90; OR = 1.71, 95% CI 1.37–2.14; and OR = 1.37, 95% CI 1.06–1.77, respectively]. In addition, participants with symptoms of DED were more likely to experience composite factors related to mental health (OR = 1.91, 95% CI 1.07–3.39).

**Table 3:** Odds ratio (OR) and 95% confidence interval (CI) for a multiple logistic regression analysis of associations between mental health-related characteristics and symptoms of DED.

	Crude OR		Adjusted OR					
			Model 1^a^		Model 2^b^		Model 3^c^	
	OR	95% CI	OR	95% CI	OR	95% CI	OR	95% CI
**Sleep duration (h/night)**								
≤ 5	1.34^†^	1.16–1.55	1.23^†^	1.06–1.42	1.27^†^	1.09–1.47	1.42^†^	1.06–1.90
6	1.12	0.97–1.28	1.14	0.99–1.32	1.16^†^	1.01–1.34	1.25	0.93–1.69
7	1	Reference	1	Reference	1	Reference	1	Reference
8	0.99	0.86–1.14	0.97	0.85–1.12	0.97	0.85–1.12	0.95	0.71–1.28
≥ 9	1.03	0.83–1.30	0.97	0.78–1.23	0.92	0.72–1.16	0.99	0.62–1.52
**Psychological stress perception (yes)**	1.45^†^	1.30–1.63	1.40^†^	1.25–1.57	1.43^†^	1.27–1.60	1.71^†^	1.37–2.14
**History of depressed mood (yes)**	1.43^†^	1.25–1.64	1.24^†^	1.08–1.42	1.28^†^	1.11–1.47	1.37^†^	1.06–1.77
Composite factors related to mental health* (yes)	1.82^†^	1.34–2.48	1.54^†^	1.12–2.10	1.65^†^	1.20–2.28	1.91^†^	1.07–3.39

^a^Model 1: Adjusted for age and gender.

^b^Model 2: Model 1 + adjusted for lifestyle factors (education, occupation, body mass index, smoking, regular exercise, and daily sun exposure).

^c^Model 3: Model 2 + adjusted for medical factors (pregnancy, thyroid disease, and history of ocular surgery).

*Composite factors related to mental health; short sleep duration (≤ 5 h/night) + psychological stress perception (yes) + history of depressed mood (yes).

^†^*P* < 0.050.

## Discussion

Upon exploring the correlation between symptoms of DED and mental health-related factors among Koreans aged ≥ 20 years, this study showed that subjects with symptoms of DED were more likely to experience short sleep durations (≤ 5 h/night), psychological stress perception, and a history of depressed mood. This result is consistent with previous studies. In addition, this study also showed that subjects with symptoms of DED were most likely to experience composite factors related to mental health [combined short sleep duration (≤ 5 h/night), psychological stress perception (yes), and history of depressed mood)].

DED is a common irritating eye disease that causes pain, discomfort, burning, foreign body sensations, and blurred vision. Previous studies have evaluated the association between psychological problems and eye diseases such as age-related macular degeneration (AMD), cataract, glaucoma, and DED^19^. DED is associated with mental health issues such as depression and anxiety^20^. Wan et al.^20^ found that the prevalence of depression and anxiety was greater in patients with DED than in the control group. Moreover, the prevalence and severity of depression were highest in patients with primary Sjögren’s syndrome, which was similar to the results obtained in this study. A meta-analysis evaluating the correlation between eye disease and depression showed that the prevalence of depression among patients with DED was 29%, 25% among patients with glaucoma, 24% among patients with AMD, and 23% among patients with cataract, showing the highest prevalence among patients with DED^19^.

Depression is more closely associated with dry eye symptoms than with dry eye signs^21,22^. Irritation of the ocular surface exerts a negative effect on visual performance and daily activity^23^. Kitazawa et al.^24^ evaluated the association between psychiatric symptoms and objective measures (break-up time [BUT], Schirmer value, and fluorescein staining score) in 56 DED patients. To quantify psychiatric symptoms, the Montgomery-Asberg Depression Scale (MADRS) and HAM-A (Japanese version of the Hamilton Rating Scale for Anxiety) scales were used. Subjects were divided into those with normal MADRS and HAM-A scores, and those with high MADRS and HAM-A scores, to compare the differences in the subjective symptoms and the objective measures of DED. There was a significant difference in the subjective symptoms of DED, whereas no significant difference was observed in the objective measures.

Galor et al.^25^ stated that patients with depression and anxiety can experience central sensitization, which may affect pain perception. In this regard, patients with depression or anxiety may react more sensitively to ocular sensation than control participants^20^ and experience persistent irritation from eye dryness, potentially causing or exacerbating mood disorders^26^. Furthermore, inflammatory cytokines play an important role in the pathogenesis of depression by triggering sickness behavior^27,28^. Lymphocytes of patients with depression activate the production of IL-1b, IL-6, and TNF, and such inflammatory pathways are known to cause neuronal damage through oxidative and nitrosative stress^27,28^. Mrugacz et al.^29^ explored the correlation between inflammatory cytokine levels in tears and depression among 32 (14 male, 18 female) DED patients with an average age of 44.21 years; and observed that patients with depression had significantly higher inflammatory cytokine (IL-6, IL-17, and TNF-α) levels in tears than control participants, which reflects the severity of local immunological changes.

Thus far, little is known about the pathogenesis of DED, and it is unclear whether depression exacerbates DED. Ayaki et al.^18^ hypothesized that DED causes distress, which can lead to depression and sleep disorders. Furthermore, eye pain and exposure to light resulting from DED can cause sleep disturbances, which can in turn lead to sleep disorders. Yu et al.^30^ evaluated the relationship between dry eye and sleep quality in multiple dimensions in a large community-based study. Sleep quality was evaluated on multiple levels, including the Chinese version of the Pittsburgh Sleep Quality Index (CPSQI). They found that worse CPSQI scores were associated with higher Ocular Surface Disease Index (OSDI) severity and worse OSDI scores. Magno et al.^31^ evaluated the relationship between dry eye and sleep quality. The results showed that patients with dry eye were one-and-a-half times more likely to be poor sleepers, with worse outcomes in all components of the PSQI. Lee et al.^16^ evaluated the association between sleep duration and dry eye syndrome (DES) in Korean adults. They showed that the prevalence of DES was 1.20 and 1.29-fold higher in the short sleep duration (5 h/day) and severe sleep disruption (≤ 4 h/day) groups, respectively, than in the optimal sleep group (6–8 h/day), showing that the prevalence of DES increased with shorter sleep duration. This is thought to be due to stress from sensory discomfort or optical disturbance experienced by patients with DED, as well as sleep difficulty from inflammatory processes, including pain and brightness due to incomplete eye closure, during sleep.

Irritating ocular symptoms can exert a negative influence on visual performance^32^ through tear instability from DED, which leads to blurred vision, thereby potentially hindering daily activities. Furthermore, patients with DED can experience stress from sensory discomfort or optical disturbances, and the condition itself can cause psychological distress. Patients with Sjögren’s syndrome are known to experience various sleep disorders such as pain, night awakenings, long sleep latency, and obstructive sleep apnea^18^.

It remains unclear whether poor sleep causes DED or vice versa. In this study, it was found that people who had a short sleep duration, experienced psychological stress perception, and reported a history of depressed mood had more symptoms of DED than when they had each single element depression in 301 patients with DED and 202 with other ocular surface diseases (chronic conjunctivitis/allergic conjunctivitis). They showed that the mild to severe DED group had a higher proportion of patients presenting with sleep quality problems, based on a Pittsburgh Sleep Quality Index (PSQI) of ≥ 6, as well as depression symptomology, based on a Hospital Anxiety and Depression Scale (HADS) of ≥ 5, when compared with the chronic conjunctivitis or allergic conjunctivitis groups. Li et al.^33^ reported that sleep deprivation in a mouse model impaired the function of the lacrimal system and caused dry eye. Lee et al.^34^ also reported that sleep deprivation could trigger dry eye by inducing tear hyperosmolarity and reducing tear secretion. Conversely, it has been shown that chronic pain and discomfort caused by DED can negatively affect sleep quality and mental health^35^. Moreover, quality of sleep improved after topical treatment for newly diagnosed DED^35^. Therefore, ophthalmologists may report difficulties in sleep and mental health together when treating patients with DED.

In some patients with DED, tear production may have been compromised by the use of somnifacients containing anticholinergic agents^36^. Chronic exposure to histamines and 5-hydroxytryptamine changes function of secretory processes, and the neuronal release of 5-hydroxytryptamine could plausibly affect the acute control of lacrimal secretion^36^. Zheng et al.^19^ stated that chronic pain from dry eye disease can induce depression, and that medications used to treat depression can cause or exacerbate dry eye disease. Additionally, sleep deprivation and medication history due to psychiatric disorders may partially inhibit tear secretion since lacrimal secretion is under neural regulation. Notably, Ayaki et al.^32^ found that steroid or mucin secretagogue eye drops (diquafosol and rebamipide) used to relieve severe DED symptoms could cause distress.

In addition, there is a possibility that coexisting autoimmune-related comorbidities (autoimmune diseases, psychiatric disorders, and chronic pain syndromes) may affect the relationship between dry eye and sleep. Magno et al.^31^ found that one in two patients with severe dry eye syndrome had chronic diseases, such as osteoarthritis and obstructive sleep apnea syndrome, that could impair sleep quality. Accordingly, the possibility of comorbidities of dry eye should be considered when consulting patients with DED.

A major strength of this study is that it evaluated DED in people who experienced sleep problems, psychological stress, and depressive moods. In addition, this study included 16,471 South Korean adults aged ≥ 20 years. Korea is a single-race country with uniform genetic and environmental influence. Therefore, the results of this study are more consistent than other large population-based studies and should be broadly generalizable.

However, the following limitations should be considered. First, although a cross-sectional study can identify the correlation between DED and psychiatric factors, it cannot establish a causal relationship. Longitudinal studies are required to confirm the associations observed in this study. Second, the DED and mental health variables used in the present study did not undergo an objective test, and it is not known whether the participants currently have dry eye. Nevertheless, the same diagnostic criteria for DED and mental health have been applied in previously reported studies^9,12^. In addition, according to a survey conducted by the Korean Corneal Disease Study Group, Korean corneal specialists diagnosed DED using the diagnostic criteria of the International Dry Eye Workshop or Dysfunctional Tear Syndrome Study Group guidelines. There is evidence that it has been tested on an accepted standard^37^. Third, sleep duration was evaluated through surveys. Fourth, we did not examine recent data beyond 2010 to 2012. Although many dry eye studies have been reported, few studies have been conducted using population-based national data, which formed the basis of our study. Future studies with objective evaluations of sleep quality (actigraphy, melatonin measurement, and polysomnography) in patients with DED are needed to further elucidate the role of mental health-related factors in the symptoms of DED.

In conclusion, the present study provides epidemiologic evidence that DED is associated with sleep duration, stress, and depression in the Korean population. Short sleep duration (≤ 5 h/night), psychological stress perception, and history of depressed mood were significantly associated with DED, even after controlling for demographic, lifestyle, and medical factors. Subjects with symptoms of DED were most likely to experience composite factors related to mental health [combined short sleep duration (≤ 5 h/night), psychological stress perception (yes), and history of depressed mood)]. Therefore, it is likely that ophthalmologists may report difficulties in sleep and mental health when treating patients with DED. In addition, psychological support and sleep treatment should be considered together with DED treatment.

## Patients and methods

### Study design and population

The Korean National Health and Nutrition Examination Survey (KNHANES) is a nationwide population-based cross-sectional health examination and survey conducted regularly by the Korea Centers for Disease Control and Prevention, under the Ministry of Health and Welfare. The fifth KNHANES survey was conducted between 2010 and 2012. This study used a complex, stratified, multistage probability sampling model based on national census data. Survey sample weights were used in all analyses. This was calculated by considering the sampling rate, response rate, and age/gender ratio of the reference population to provide a representative estimate of the Korean civilian population. Previous studies have described the KNHANES methodology in detail^37–40^. In addition, survey datasets are freely available on the KNHANES website.

In total, 31,596 people participated in the fifth KNHANES V, representing 11,400 households (3800 households/year) across 576 national districts (192 national districts per year). The response rates for the 2010, 2011, and 2012 KNHANES were 81.9% (n = 8958), 80.4% (n = 8518), and 80.0% (n = 8058), respectively (Fig. 1).

Participants in the survey underwent a health interview comprising health examinations, an ophthalmic interview and ophthalmic examinations, and a nutrition survey. A health interview and examination were performed by trained interviewers and medical staff at the mobile examination center. The ophthalmic survey was verified by the Epidemiology Survey Committee of the Korean Ophthalmological Society, and the detailed diagnostic criteria for eye conditions used in this study have been described elsewhere^12^.

This study followed the tenets of the Declaration of Helsinki for biomedical research and was approved by the Institutional Review Board (IRB) of the Korea Center for Disease Control and Prevention (KCDC) (No: 2010-02CON-21-C, 2011-02CON-06-C, 2012-01EXP-01-2C). Written informed consent was obtained from all the participants during the 2010–2012 KNHANES.

### Participants and data selection

We included 25,534 participants who completed the KNHANES 2010–2012. We excluded participants aged < 19 years (n = 6140), or those who had no questionnaire related to DED (n = 2599). We further excluded those without mental health characteristics (n = 324). Finally, after implementing these exclusion criteria, 16,471 participants were included in the analysis (Fig. 1).

The DED questionnaire used in this study was designed by the Korean Ophthalmology Society (KOS). For quality control, ophthalmologists are trained twice a year by the Korean Disease Control and Prevention Agency (KCDC) and KOS. The KNHANES introduced dry eye questionnaires in 2010 to evaluate the prevalence of dry eyes^37^. DED was determined based on the symptoms using a questionnaire administered by an ophthalmologist. The following questions were answered “yes” or “no”: “Do your eyes tend to dryness, foreign body sensation with itching and burning or sandy feeling lately?” Asking about the symptoms of DED is a reliable diagnostic method, as there are currently no definitive clinical diagnostic tests to diagnose an individual with DED.

Sleep duration was assessed using the self-administered question, “How many hours do you usually sleep a day?” The third edition of the International Classification of Sleep Disorders generally classifies long sleepers as those sleeping for ≥ 9–10 h, and short sleepers as those sleeping for ≤ 5–6 h^41,42^. Therefore, this study classified sleep time as ≤ 5, 6, 7, 8, or ≥ 9 h/day, according to the intervals used in a previous study^43^.

Psychological stress perception was evaluated from responses indicative of cognitive complaints collected with a questionnaire using a 4-point Likert scale [1 (very much), 2 (a lot), 3 (a little), and 4 (hardly any)] to the question “How much stress do you feel in your daily life?”^44^. Psychological stress perception was classified as “yes” when the level of stress perception was very much or a lot and “no” when a little or hardly any. Participants were asked to respond “yes” or “no” to the following history of depressed mood questions: “During the past year, has your daily life been burdened by feelings of hopelessness or dejection for more than 2 continuous weeks?”^9^. Participants were defined as having a composite factor related to mental health when they answered positively regarding sleep duration (≤ 5 h/night), psychological stress perception (yes), and history of depressed mood (yes).

Questionnaires were used to collect data on age, sex, education, household income, occupation, smoking, regular exercise, daily sun exposure, pregnancy, and a history of ocular surgery. Subjects were classified into six age groups: 20–29, 30–39, 40–49, 50–59, 60–69, and ≥ 70 years. Educational attainment was classified into the following categories: less than elementary school education, middle school, high school, and more than university. Household income was then divided into quartiles. Occupation was classified as blue collar (agriculture workers, forestry workers, fishery workers, craft and related trade workers, plant and machine operators and assemblers, and simple labor), white collar (managers, professionals, clerks, and service or sales workers), and unemployed for any reason. Current smoking was classified as yes or no (current non-smokers were either never smokers or past smokers). Regular exercise was defined as walking for > 30 min at least 5 days a week. Daily sun exposure was classified into categories of < 2, 2–5, and ≥ 5 h of typical exposure per day. Pregnancy was assessed using the question “Are you currently pregnant?” History of ocular surgery was evaluated using the question, “Have you ever had ocular surgery in the past?”.

Anthropometric measurements were used to collect data on body mass index (BMI), diabetes, hypertension, thyroid disease, and rheumatoid arthritis. BMI was defined as underweight (< 18.5 kg/m^2^), normal (≤ 18.5 to < 25 kg/m^2^), and overweight/obesity (≥ 25 kg/m^2^)^43^. Diabetes was evaluated using the question: “Have you ever diagnosed with diabetes by a physician?” Hypertension was defined as a systolic blood pressure > 160 mmHg and/or diastolic blood pressure > 90 mmHg, measured during the medical examination, or currently taking antihypertensive medications^43^. Thyroid disease and rheumatoid arthritis diagnoses were classified into combined yes or no categories using the question: “Have you ever been diagnosed with thyroid disease or rheumatoid arthritis by a physician?”.

### Statistical analyses

Weighted analysis was used to reflect the true population statistics more accurately. Data are expressed as unweighted numbers and weighted proportions (%). The chi-square test was used to compare the demographic, lifestyle, and medical characteristics of the study population between participants with and without DED symptoms. Multiple logistic regression analysis was conducted to examine the odds ratios (ORs) and 95% confidence intervals (CIs) for the association between mental health-related characteristics (sleep duration, psychological stress perception, and history of depressed mood) and the symptoms of DED. Demographic factors (age and sex), lifestyle factors (education, occupation, BMI, smoking, regular exercise, and daily sun exposure), and medical factors (pregnancy, thyroid disease, and history of ocular surgery) were used as covariates to calculate the adjusted odds ratios (aORs). Statistical analyses were performed using SPSS 18.0 version (SPSS Inc., Chicago, IL, USA). All reported *P* values were two-tailed, and values < 0.050 were considered as the threshold for statistical significance.

## Data Availability

All data supporting the conclusions of this study are included in the present article.
